# Scale Validation Conducting Confirmatory Factor Analysis: A Monte Carlo Simulation Study With LISREL

**DOI:** 10.3389/fpsyg.2018.00751

**Published:** 2018-05-22

**Authors:** Daniel Ondé, Jesús M. Alvarado

**Affiliations:** Psychobiology and Behavioral Sciences Methods, Complutense University of Madrid, Madrid, Spain

**Keywords:** Monte Carlo simulation study, confirmatory factor analysis, maximum likelihood, unweighted least squares, goodness-of-fit indices, LISREL

## Introduction

When psychologists are going to test their theoretical models (at the time of planning the research study), several questions may arise regarding the quality and potential accuracy of the estimation of Confirmatory Factor Analysis (CFA) models under certain applied conditions. For example, one question is the minimum sample size (*N*) and/or the number of indicators per factor (*p*/*k*) that is needed to estimate the CFA models properly. Many of these questions can be answered through simulation studies, because the magnitudes of the population factor loadings (λ_*ik*_) are known in advance. Monte Carlo simulation uses random sampling and statistical modeling to estimate mathematical functions, and is a key tool for studying analytically intractable problems (Harrison, 2010). It is quite frequent to find in the literature simulation studies that use CFA to fit measurement models. However, there is a lack of technical information in the published research to replicate this type of studies, probably due to length limitations. Furthermore, researchers and scholars must often go to numerous and technically complex sources of information to understand the laborious simulation and estimation process.

In this paper we present complete technical information to conduct Monte Carlo simulation CFA-studies with PRELIS and LISREL programs. The LISREL program, apart from being one of the most used (and validated) software programs, is historically linked to CFA (Brown, 2015). Although it is a commercial program, there is a free student version that allows performing all the simulation tools and the analysis techniques shown in this work. Through a simulation study, we have evaluated the necessary conditions to test the CFA models fit, so we have chosen population structures formed by a single common factor to design the simulation study and low-moderate population factor loadings. It should be noted that these types of studies generate a considerable volume of information, even when using a simple unifactorial model. This approach was adopted for this study for ease of understanding the principles. Included, however, are the PRELIS and LISREL syntax examples for multidimensional factor structures. In the Supplementary Material (https://figshare.com/s/18eb0e998150d39bc952) we have attached both the simulated data and the CFA results (parameter estimates, standard errors and goodness-of-fit measures).

## Monte carlo simulation study

### Simulated conditions

The simulated experimental conditions reflect low-medium sample sizes (*N* = 200, 300, 400, and 500), low-moderate model sizes (indicators per factor: *p*/*k* = 4, 5, 6, 7, and 15), and low-moderate population factor loadings (λ_*ik*_ = 0.2, 0.3, and 0.4). The unifactorial structures simulated are equal-λ_*ik*_ condition (i.e., all *p*/*k* indicators of each population structure have the same λ_*ik*_ magnitude). The selected sample-model size is frequent in applied research. For example, after reviewed 1,409 published CFA models, Jackson et al. (2009) found a median *N* of 389. On the other hand, a common recommendation in some applied context is not to interpret estimated factor loadings < 0.30 (Brown, 2015).

### Data generation

The data generation process was conducted using PRELIS 2 program (Jöreskog and Sörbom, 1996b), according to the common factor model showed in Equation (1):

(1) $$\begin{array}{l} \Sigma =\Lambda \Phi \Lambda^{\prime }+\Theta \end{array}$$

were **Σ** is the population correlation matrix, **Λ** is the population factor loading matrix, **Φ** is the population factor correlation matrix, and **Θ** is the unique variances matrix. All indicators were simulated as continuous variables, computed by the expression shown in Equation (2):

(2) $$\begin{array}{l} x=\Lambda \xi +\delta \end{array}$$

Equation (2) summarized a set of equations (one for each indicator: ***X***_*i*_ = **λ**_*ik*_ **ξ**_*k*_ + **δ**_*i*_) that express the relationship among the observed variables (***X***_*i*_), the common factor (**ξ**_*k*_) and the unique variances (**δ**_*i*_).

Step 1 of the data generation process has consisted, following Equation 2, in the simulation of the common factor (**ξ**_*k*_) as a random variable (in this case, **ξ**_*k*_ = 1, see Jöreskog and Sörbom, 1996b; Brown, 2015), and the computation of the *p*/*k* indicators. Following the recommendation of Kelley and Pornprasertmanit (2016), we have simulated 1,000 normal distributed data matrix **X** (order *N* x *p*/*k* indicators), with the same integer starting value for the random number generator in all the simulated conditions (see Example 1 from Presentation 1). To extend the experimental simulation conditions, Example 1 shows how to recode continuous variables into discrete variables using thresholds, and how to simulate non-normal distributed indicators.

For each data matrix **X** generated in step 1, step 2 has consisted in the generation of files that contain variance-covariance matrices (**S**) and files that contain correlation matrices (**R**). That is, both types of **R** and **S** files come from the same raw data (see Example 2 from Presentation 1).

### Estimation of CFA model parameters

The objective of CFA is obtaining estimates for each parameter of the measurement model and computing a predicted variance-covariance or correlation matrix (Σ^*^) that best reproduces the observed or input matrix (**S** or **R**). In practice, perfect fit is not possible due to the measurement error, so the starting point of analysis implies that Σ^*^ ≠ **S** or **R** (Bollen, 1989). The residuals (*d*_*i*_) are the differences between each pair of predicted-observed covariances or correlations, so vector **d** = {*d*_1_*, d*_2_*,…, d*_*i*_} indicates the degree of discrepancy between both matrices. The estimation process consists in an iterative numerical technique to minimize the discrepancy between Σ^*^ and **S** (or **R)** matrices (Bollen, 1989; Brown, 2015). This numerical technique is a lineal fitting function [also called discrepancy function *F*(**S**; Σ^*^) or *F*(**R**; Σ^*^)], which takes different forms depending on the estimation method used.

Maximum Likelihood (ML) fitting function is widely used in applied CFA research (Brown, 2015). ML minimization function can be expressed as *F*_ML_ = **d'Wd** (where **W** is a weighted matrix), where *F* is calculated from each computed vector **d** at each step of the iterative process. Unweighted Least Square (ULS) is an easy to understand fitting function, *F*_ULS_ = **d'd**, where *F* is obtained by calculating the sum of squares of the residuals *d*_1_*, d*_2_*,…, di* (**W** can be replaced by an identity matrix (**I**), simplifying the ML discrepancy function). *F*_ULS_ estimates are consistent estimates with sufficient sample size, although less efficient than *F*_ML_ estimates (Bollen, 1989).

A cautionary note should be made regarding the use of **S** and **R** matrices. The general rule is that **S** should be analyzed, although a common practice is analyzing **R** as if they were **S** matrices (Jöreskog and Sörbom, 1996a, p. 35). However, as Cudeck (1989) pointed out, statistical theory for the analysis of CFA structures has been most completely developed for applications to **S**, and the analysis of **R** may be problematic in many situations. The analysis of **R** with ML may modify the model that is being analyzed, may product incorrect χ^2^ and derived indices, and may give incorrect standard errors (Jöreskog et al., 2001). The only advantage of **R** is improving the interpretability of the solutions. When variables have quite different variances, may be useful analyzing **R** matrices. This is a common practice when fitting a regression models or using an Exploratory Factor Analysis (Cudeck, 1989, pp. 325–326). The general recommendation is to use ML estimation method with **S** and ULS with **R** (for a more detailed description, see Bollen, 1989, pp. 104–113). In addition, when a **S** matrix is analyzed, the completely standardized solution can be obtained, so it is no necessary to analyze **R** to obtain a more interpretable solution. Note that a **R** matrix is a completely standardized **S** matrix (Brown, 2015). A full description of how analyzing polychoric/tetrachoric matrices, with PRELIS/LISREL syntax examples, can be found in Yang-Wallentin et al. (2010).

Example 3 from Presentation 1 shows LISREL syntax (Jöreskog and Sörbom, 1996a) to estimate CFA models from the simulated dataset. A convergence criterion equal to 250 has been fixed in all the simulated conditions.

## Dataset overview and outputs

The Supplementary Material includes a set of files with simulated raw-sample data from all the experimental conditions. The name of each file has two parts. The letter “N” denotes the sample size used to generate the data collected by each file (N200, N300, N400, and N500), and L020, L030 and L040 denote the simulated (λ_*ik*_) magnitude (0.2, 0.3, and 0.4, respectively). There are 24 SPSS/Excel files with raw data, (e.g., for N = 200: N200L020, N200L030, N200L040). In addition, we present the estimated solutions conducting CFA with LISREL program from the proposed simulation study. An overview of all of this information and outputs can be consulted in Presentation 2, and the results of each estimation process can be examined in two dataset:

- Continuous_data_INFORMATION.pdf.
- Continuous_Data_Normal_ML.
- Continuous_Data_Normal_ULS.

The last two types of files contain the parameter estimates, the standard errors and different goodness-of-fit measures. The analysis of **S** with ML generate unstandardized solutions (Brown, 2015). The unstandardized parameter estimates can be consulted in Unstandardized_parameters_ML files. To facilitate the direct comparison between ML and ULS estimators, ML files contain standardized solutions (default option in analysis of **R**). Note that LISREL provides standard errors for each estimated parameter in the unit of measurement of the indicators (Bollen, 1989; Jöreskog and Sörbom, 1996a). Therefore, to construct statistical tests (*t*-test) for estimated factor loadings ($\lambda_{ik}^{*}$) with the parameter estimates provided by ML method, the unstandardized values must be used.

Coefficient of congruence (see Equation 3) was computed as an index of factor similarity:

(3) $$\begin{array}{l} C_{k}=\frac{\Sigma_{i=1}^{p}\lambda_{ik}^{*}\lambda_{ik}}{\sqrt{\left(\Sigma_{i=1}^{p}\lambda_{ik}^{*2}\right)\left(\Sigma_{i=1}^{p}\lambda_{ik}^{2}\right)}} \end{array}$$

This coefficient computes the discrepancy between λ_*ik*_ and λ^*^_ik_ for each indicator *i* of factor *k*, where *p* is the number of items per factor (*p*/*k*), and reflects a combined measure of good or poor parameter recovery of a given cluster of indicators. Lorenzo-Seva and Ten Berge (2006) have shown that congruence values in the range of 0.85–0.95 can be considered as “fair similarity” between λ_*ik*_ and λ^*^_ik_, and values higher than 0.95 as “good similarity”.

Through the study shown in this work, the researchers can use some of the outputs attached in the Supplementary Material to evaluate different general issues related to the potential quality and accuracy of the CFA models. Two applications are described below:

*Application 1*: The researchers can explore the accuracy of parameter estimation, and the statistical relationship between the parameter recovery (*C*_*k*_), the overall model fit (χ^2^), and a high number of goodness-of-fit indices. Note that the χ^2^ values of the output files depend on the method of estimation used to fit the model to the data (Jöreskog, 2004). Table 1 shows a descriptive summary of this type of information obtained from continuous data ML file. First, we have filtered the dataset removing the improper solutions (non-convergent solutions and Heywood cases). As can be seen, the parameter recovery (*C*_*k*_) improves as λ_*ik*_, *p*/*k*, and *N* increase.

**Table 1:** Descriptive summary of estimated CFA solutions (ML method): rate of proper solutions, parameter estimates (factor average), coefficient of congruence, RMSEA and CFI [%, Mean, (SD), min and max].

***λ_*ik*_***		**4–6 indicators**		**7 indicators**		**15 indicators**	
		***N* = 200–300**	***N* = 400–500**	***N* = 200–300**	***N* = 400–500**	***N* = 200–300**	***N* = 400–500**
0.2	Proper solutions (%)	62.9%	72.5%	75.8%	88.7%	98.1%	100%
	Average λ^*^_ik_	0.24 (0.04)	0.22 (0.03)	0.22 (0.03)	0.21 (0.03)	0.20 (0.02)	0.20 (0.02)
	Average λ^*^_ik_ (min-max)	0.14–0.38	0.10–0.35	0.13–0.32	0.12–0.30	0.13–0.28	0.11–0.26
	*C_*k*_*	0.84 (0.09)	0.86 (0.09)	0.84 (0.09)	0.87 (0.08)	0.88 (0.06)	0.93 (0.03)
	RMSEA	0.01 (0.02)	0.01 (0.02)	0.01 (0.02)	0.01 (0.01)	0.01 (0.01)	0.01 (0.01)
	CFI	0.70 (0.42)	0.82 (0.33)	0.78 (0.35)	0.86 (0.26)	0.85 (0.22)	0.93 (0.10)
0.3	Proper solutions (%)	93.0%	98.9%	99.1%	100%	100%	100%
	Average λ^*^_ik_	0.31 (0.04)	0.30 (0.03)	0.30 (0.03)	0.30 (0.02)	0.30 (0.02)	0.30 (0.02)
	Average λ^*^_ik_ (min-max)	0.15–0.44	0.17–0.42	0.20–0.41	0.20–0.38	0.22–0.38	0.25–0.35
	C_*k*_	0.92 (0.06)	0.96 (0.04)	0.94 (0.04)	0.97 (0.02)	0.97 (0.02)	0.98 (0.01)
	RMSEA	0.01 (0.02)	0.01 (0.02)	0.01 (0.02)	0.01 (0.01)	0.01 (0.01)	0.01 (0.01)
	CFI	0.94 (0.15)	0.97 (0.08)	0.95 (0.09)	0.97 (0.05)	0.97 (0.05)	0.98 (0.02)
0.4	Proper solutions (%)	99.8%	100%	100%	100%	100%	100%
	Average λ^*^_ik_	0.40 (0.04)	0.40 (0.03)	0.40 (0.03)	0.40 (0.02)	0.40 (0.02)	0.40 (0.02)
	Average λ^*^_ik_ (min-max)	0.25–0.52	0.28–0.50	0.28–0.50	0.32–0.47	0.33–0.48	0.35–0.45
	*C_*k*_*	0.97 (0.02)	0.99 (0.01)	0.98 (0.01)	0.99 (0.01)	0.99 (0.01)	0.99 (0.00)
	RMSEA	0.02 (0.03)	0.01 (0.02)	0.02 (0.02)	0.01 (0.01)	0.01 (0.01)	0.01 (0.01)
	CFI	0.98 (0.06)	0.99 (0.04)	0.98 (0.03)	0.99 (0.02)	0.99 (0.02)	0.99 (0.01)

**Figure 1:**
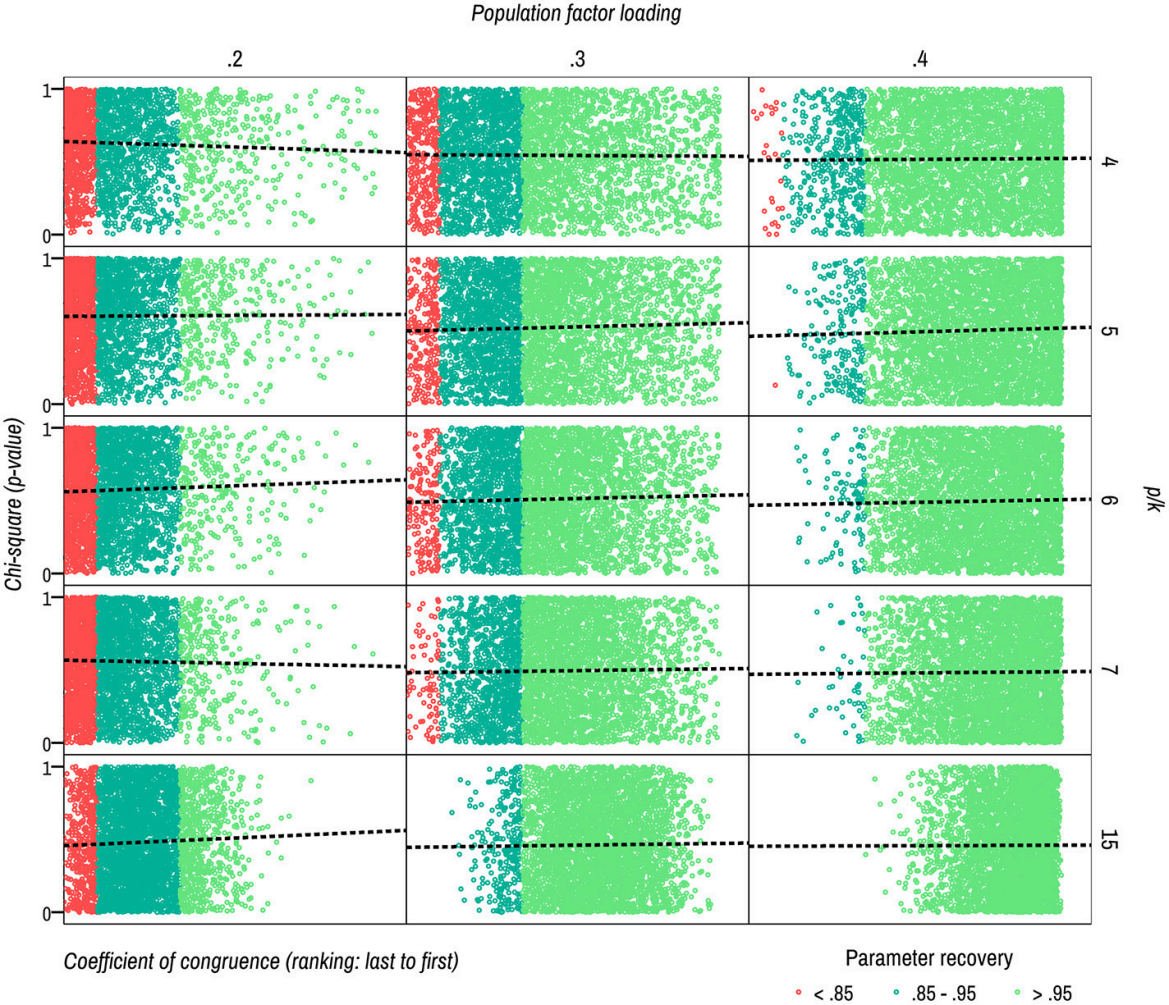
Scatter plots for parameter recovery (x-axis) and overall fit (y-axis). ML estimation. Ranking of coefficient of congruence show the relative position of *C*_*k*_ values from all estimated solutions (ordered from solution with lowest *C*_*k*_ -last position- to higher *C*_*k*_ -first position-).

Table 1 shows that several of the estimated solutions present a certain degree of overestimation or underestimation of the parameters (λ^*^_ik_). A better understanding of the conditions in which this lack of precision occurs is an important issue. Especially in the case of overestimation, the applied researchers may be too optimistic in the theoretical interpretation of the evaluated models. Moreover, Table 1 also shows that the Root Mean Square Error (RMSEA) are not informative of this lack of accuracy (Heene et al., 2011). The Comparative Fit Index (CFI) is more informative in the most suboptimal conditions (i.e., λ_*ik*_ = 0.2). This situation may be contributing to confuse the theoretical interpretations of some applied researchers. Additionally, there are no relationship between accuracy of the parameter recovery (*C*_*k*_) and χ^2^ *p*-value, as can be seen in Figure 1.

Researchers and scholars can reuse these databases in order to explore in depth the conditions under which over-underestimation of the parameters may occur, or to evaluate the performance of the alternative goodness-of-fit indices. They can also reuse the provided syntax examples in order to extend the number and complexity of the experimental conditions.

*Application 2*: Each dataset provided in Supplementary Material has a “CASE” variable. This variable allows the raw data location of each CFA estimated solution collected in the two main output files, or a specific unstandardized solution for ML estimation, and it can be useful to review in depth the internal structure of certain estimated CFA models. For example, CASE = 90 (λ_*ik*_ = 0.3, *p*/*k* = 4, and *N* = 200) results in an improper solution when the ML estimation method is used. There are many situations that can lead to an improper solution when a CFA model is fitted to the data (Brown, 2015). The condition of positive definiteness input matrix can be evaluated by Principal Component Analysis (PCA). If all eigenvalues are greater than zero, as in CASE = 90, the matrix is positive definite (Wothke, 1993). Then, we have explored the effect of outliers over estimation process. We have computed a new normally distributed random variable in the CASE = 90 raw dataset, that has been used as a criterion variable in a regression analysis with the *X*_1_ to *X*_4_ indicators as predictors. This analysis has helped us to identify outliers in the data (from Mahalanobis distances that are statistically significant at 0.05). Once these cases have been removed from raw data, CFA analysis produced an estimable-proper solution with *C*_*k*_ = 0.863.

## Conclusion

Following open science philosophy (Munafò et al., 2017), in this paper we have shown how to estimate unifactorial models conducting CFA from a Monte Carlo simulation study with PRELIS and LISREL programs, which are some of the most used in Structural Equation Modeling (SEM) and CFA analysis. Additionally, we have made some indications about the estimation of CFA multidimensional models, with syntax examples, in order to facilitate the generalization of CFA analysis in more complex population structures. The applications presented are not intended to be an exhaustive evaluation of the results obtained, but rather an exposition of the possibilities of the data generated and the estimation process implemented that may be of interest for academic and research purposes.

## Author contributions

DO and JA developed the idea together. DO conducted the Monte Carlo simulation study with PRELIS/LISREL programs and collect all outputs into different dataset. JA supervised all the simulation process. DO drafted the manuscript and all authors approved the final version after discussing the intellectual content. All authors agreed to be accountable for all aspects of the work.

### Conflict of interest statement

The authors declare that the research was conducted in the absence of any commercial or financial relationships that could be construed as a potential conflict of interest.

## Supplementary material

### Data availability

In the Supplementary Material (https://figshare.com/s/18eb0e998150d39bc952) we have attached both the simulated data and the CFA results (parameter estimates, standard errors and goodness-of-fit measures).
