# Supplementary material for: Scale Validation Conducting Confirmatory Factor Analysis: A Monte Carlo Simulation Study With LISREL
Source: Front Psychol. 2018 May 22;9:751. doi: 10.3389/fpsyg.2018.00751 (PMC5972281; doi:10.3389/fpsyg.2018.00751)
Supplement: Supplementary file 1 [file Presentation_1.pdf]

Supplementary Examples

Example 1 (PRELIS)

```
DA NO=200 RP=1000
NE F1=NRAND
NE V1=0.4*F1+0.9165*NRAND
NE V2=0.4*F1+0.9165*NRAND
NE V3=0.4*F1+0.9165*NRAND
NE V4=0.4*F1+0.9165*NRAND
SD F1
CO ALL
OU RA='[disc]:\[folder1]\[folder2]\...\[filename].DAT' WI=7 ND=3 XB XM XT XO IX=999
```

NOTES:

1. Simulation conditions: p/k = 4, N = 200 and λ_{ik} = .4.
2. Line NE F1=NRAND is used to generate the common factor (ϕ_{11}) as a random variable.
3. To simulate X_i with λ_{ik} = .4, we compute NE V_i=0.4*F1+0.9165*NRAND, where error term (δ_i) is equal to $(1 - \lambda_{i1}^2)^{½}$ *NRAND
4. NRAND expression can be replaced by NRAND**2+NRAND**2 to obtain a non-normal indicator (~χ^2 with 2 *df*, or can be replaced by CRAND(2).
5. To recode continuous indicators into discrete indicators using thresholds, insert line with RE command (after SD F1 line), and replace CO option by OR option (replacing “continuous” by “ordinal”). For example, using following lines allows to generate 4 ordinal variables with 5 symmetric response categories:

```
RE V1-V4 OLD=-100 - -1.64485,-1.64484 - -0.67448,-0.67447 - 0.67448,0.67449 - 1.64485,1.64486 - 100 NEW=1,2,3,4,5
OR ALL
```

Example 2 (PRELIS)

```
! CM for variance-covariance matrices (S)
! Correlation matrices (R): replace CM for KM
DA NI=4 NO=200 RP=1000
RA='[disc]:\[folder1]\[folder2]\...\[filename].DAT'
CO ALL
OU MA=CM CM='[disc]:\[folder1]\[folder2]\...\[filename].CM' WI=7 ND=3 XO XB XM XT
```

Example 3 (LISREL)

```
! ME=ML: Maximum Likelihood (CM files)
! ME=UL: Unweighted Least Squares (KM files)
DA NI=4 MA=CM NO=200 RP=1000
KM='[disc]:\[folder1]\[folder2]\...\[filename].CM'
MO NX=4 NK=1
FR LX(1,1) LX(2,1) LX(3,1) LX(4,1)
OU PV='[disc]:\[folder1]\[folder2]\...\[filename].PV' SV='[disc]:\[folder1]\[folder2]\...\[filename].SV'
GF='[disc]:\[folder1]\[folder2]\...\[filename].GF' XM XO SS SC ME=ML IT=250
```

NOTES:

1. The input dataset is specified by CM option (**S**) or KM option (**R**) in DA command line.
2. Note that line after DA command line begins whit KM command, regardless of whether input data CM or KM option is used. When CM option is used, the estimation method (ME) must be fixed to ML (ME = ML). When KM option is used ME must be fixed to UL (i.e., ME = UL = Unweighted Least Squares).
3. When CM dataset is analyzed, the completely standardized solution is obtained by SC option on the OU command line.
4. The estimation process generates three types of output files (see OU command line): estimated parameters (.PV files), standard errors (.SV files), and goodness-of-fit measures (.GF files). For more information, see Jöreskog & Sörbom (1996b).

Example 4 (PRELIS)

```
! Convert parameter files (PV) into data files (DAT)
DA NI=11
LA
CASE CON ADM
'LX1.1' 'LX2.1' 'LX3.1' 'LX4.1'
'TD.1' 'TD.2' 'TD.3' 'TD.4'
RA='[disc]:\[folder1]\[folder2]\...\[filename].PV' FO; ((F6.0,F3.0,F3.0)/6D13.6/2D13.6); CO ALL
OU SR='[disc]:\[folder1]\[folder2]\...\[filename].DAT'
```

Example 5 (PRELIS)

```
! Convert standard errors files (SV) into data files (DAT)
DA NI=11
LA
CASE CON ADM
'EX1' 'EX2' 'EX3' 'EX4'
'ETD1' 'ETD2' 'ETD3' 'ETD4'
RA='[disc]:\[folder1]\[folder2]\...\[filename].SV' FO; ((F6.0,F3.0,F3.0)/6D13.6/2D13.6); CO ALL
OU SR='[disc]:\[folder1]\[folder2]\...\[filename].DAT
```

Example 6 (PRELIS)

```
! Convert goodness-of-fit indices files (GF) into data files (DAT)
DA NI=47
LA
CASE CON ADM
'gl' 'MFFCHI' 'MFFCHIp' 'NCHI' 'NCHIp' 'SBCHI' 'SBCHIp' 'CorrCHI' 'CorrCHIp'
'NCP' 'NCPi' 'NCPs' 'MFFV' 'F0' 'F0i' 'F0s' 'RMSEA' 'RMSEAi' 'RMSEAs' 'RMSEA05' 'ECVImod'
'ECVImodi' 'ECVImods' 'ECVIsat' 'ECVIind' 'CHIind' 'AICind' 'AICmod' 'AICsat' 'CAICind' 'CAICmod'
'CAICisat' 'RMR' 'SRMR' 'GFI' 'AGFI' 'PGFI' 'NFI' 'NNFI' 'PNFI' 'CFI' 'IFI' 'RFI' 'CNHoelter'
RA='[disc]:\[folder1]\[folder2]\...\[filename].GF' FO;
((F5.0,F3.0,F3.0,F5.0,5D13.6)/6D13.6/6D13.6/6D13.6/6D13.6/6D13.6/6D13.6/2D13.6); CO ALL
OU SR='[disc]:\[folder1]\[folder2]\...\[filename].DAT'
```

Example 7 (SPSS)

```
* Convert output data files (DAT) into SPSS files (SAV). LXUN and DUN are unstandardized parameters (ML)
GET DATA
/TYPE=TXT
/FILE="[disc]:\[folder1]\[folder2]\...\[filename].DAT "
/FIXCASE=2
/ARRANGEMENT=FIXED
/FIRSTCASE=1
/IMPORTCASE=ALL
/VARIABLES=
/1 CASE 0-11 COMMA12.0
CON 12-23 COMMA12.0
ADM 24-35 COMMA12.0
LXUN1.1 36-47 COMMA12.5
LXUN2.1 48-59 COMMA12.5
LXUN3.1 60-71 COMMA12.5
LXUN4.1 72-83 COMMA12.5
TDUN1 84-95 COMMA12.5
TDUN2 96-107 COMMA12.5
TDUN3 108-119 COMMA12.5
/2 TDUN4 0-11 COMMA12.5.
EXECUTE.
```

Example 8 (PRELIS and LISREL)

! Syntax example to illustrate data generation of 3-factor population structures (*N* = 300) – PRELIS
! Factor correlation:

1. DA NO=300 RP=1000
2. NE F0=NRAND
3. NE F1=0.5477*F0+0.83666*NRAND
4. NE F2=0.5477*F0+0.83666*NRAND
5. NE F3=0.5477*F0+0.83666*NRAND
6. NE V1=0.7*F1+0.71414*NRAND
7. NE V2=0.7*F1+0.71414*NRAND
8. NE V3=0.7*F1+0.71414*NRAND
9. NE V4=0.7*F1+0.71414*NRAND
10. NE V5=0.7*F1+0.71414*NRAND
11. NE V6=0.5*F2+0.866*NRAND
12. NE V7=0.5*F2+0.866*NRAND
13. NE V8=0.5*F2+0.866*NRAND
14. NE V9=0.5*F2+0.866*NRAND
15. NE V10=0.4*F3+0.91651*NRAND
16. NE V11=0.4*F3+0.91651*NRAND
17. NE V12=0.4*F3+0.91651*NRAND
18. SD F0-F3
19. CO ALL
20. OU RA='[*disc*]:\[*folder1*]\[*folder2*]\...\[*filename*].DAT' WI=7 ND=3 XB XM XT XO IX=999

NOTES:

1. We generate a random variable (F0) as a second-order factor, and then we generate the three factors of the multidimensional structure (F1-F3) by the following equation: $\sqrt{(0.3)}*F0+\sqrt{(1 - 0.3)}*NRAND = 0.5477*F0+0.8366*NRAND$.

! CM for variance-covariance matrices (**S**) – PRELIS
DA NI=12 NO=300 RP=1000
RA='[*disc*]:\[*folder1*]\[*folder2*]\...\[*filename*].DAT'
CO ALL
OU MA=CM CM='[*disc*]:\[*folder1*]\[*folder2*]\...\[*filename*].CM' WI=7 ND=3 XO XB XM XT

! ME=ML: Maximum Likelihood (CM files) - LISREL
DA NI=12 MA=CM NO=300 RP=1000
KM='[*disc*]:\[*folder1*]\[*folder2*]\...\[*filename*].CM'
MO NX=12 NK=3
FR LX(1,1) LX(2,1) LX(3,1) LX(4,1) LX(5,1) LX(6,2) LX(7,2) LX(8,2) LX(9,2)
FR LX(10,3) LX(11,3) LX(12,3)
OU PV='[*disc*]:\[*folder1*]\[*folder2*]\...\[*filename*].PV' SV='[*disc*]:\[*folder1*]\[*folder2*]\...\[*filename*].SV'
GF='[*disc*]:\[*folder1*]\[*folder2*]\...\[*filename*].GF' XM XO SS SC ME=ML IT=250

! Convert parameter files (PV) into data files (DAT) - PRELIS
DA NI=30
LA
CASE CON ADM
'LX1.1' 'LX2.1' 'LX3.1' 'LX4.1' 'LX5.1' 'LX6.1'
'LX7.1' 'LX8.1' 'LX9.1' 'LX10.1' 'LX11.1' 'LX12.1'
'PHI2.1' 'PHI3.1' 'PHI3.2'
'TD1' 'TD2' 'TD3' 'TD4' 'TD5' 'TD6'
'TD7' 'TD8' 'TD9' 'TD10' 'TD11' 'TD12'
RA='[*disc*]:\[*folder1*]\[*folder2*]\...\[*filename*].PV' FO; ((F6.0,F3.0,F3.0)/6D13.6/6D13.6/6D13.6/6D13.6/3D13.6); CO ALL
OU SR='[*disc*]:\[*folder1*]\[*folder2*]\...\[*filename*].DAT'
