# Supplementary material for: Scale Validation Conducting Confirmatory Factor Analysis: A Monte Carlo Simulation Study With LISREL
Source: Front Psychol. 2018 May 22;9:751. doi: 10.3389/fpsyg.2018.00751 (PMC5972281; doi:10.3389/fpsyg.2018.00751)
Supplement: Supplementary file 2 [file Presentation_2.pdf]

CONTINUOUS DATA

SIMULATION CONDITIONS AND LIST OF VARIABLES

[Last update: April 2018]

STUDY 1: GENERAL INFORMATION

1. Monte Carlos simulation study (experimental conditions)

- **Unidimensional structures**: Monte Carlo simulation from different *unifactorial* population structures.
- **Population factor loadings** (λ_{ik}): .2, .3, and .4.
- ***Tau*-equivalent or equal-loading condition (EQ)**: all indicators of each population structure have been simulated from the same λ_{ik} magnitude.
- **Indicators per factor** (p/k): 4, 5, 6, 7, and 15.
- **Sample size** (N): 200, 300, 400, and 500.
- **Data distribution**: normally distributed data [~N(0,1)].
- **Confirmatory Factor Analysis (CFA) estimation method**: *Maximum Likelihood* (ML) *and Unweighted Least Squares* (ULS)
- **Sample replications**: 1,000 for each experimental condition.

***Study 1*:**

3(EQ λ_{ik}) x 5(p/k) x 4(N) x 2(*Estimator*: ML, ULS) x 1(*distribution*) x 1,000 =

120,000 replications (120 experimental conditions).

2. Datasest notes

- **Data presentation** (SPSS/Excel format): two main files with the estimated solutions (Continuous_Data_Normal_ML and Continuous_Data_Normal_ULS), and 12 raw data files (λ_{ik} x N – see below).
- **Experimental conditions (list of *independent variables* in main ML-ULS files)**: Estimator (“method”), λ_{ik} (“lambda”), p/k (“indicators”), and sample size (“N”). Codes:
  - **“method”**: [1] ML; [2] ULS.
  - **“lambda”**: [1] .20; [2] .30; [3] .40.

- o **“indicators”**: [1] 4; [2] 5; [3] 6; [4] 7; [5] 15.
- o **“N”**: [1] 200; [2] 300; [3] 400; [4] 500.

- **Cases order (ascending)**: 1º “method”, 2º “lambda”, 3º “indicators”, 4º “N”, and 5º “CASE”. See Table 1.

Table 1. Dataset ordered by experimental conditions (ascending)

“*method*”	“*lambda*”	“*indicators*”	“*N*”	*Case*
ML [1]	.2 [1]	4 [1]	200 [1] (first ML)	1-1,000
ML [1]	.2 [1]	4 [1]	300 [2]	1-1,000
ML [1]	.2 [1]	4 [1]	400 [3]	1-1,000
ML [1]	.2 [1]	4 [1]	500 [4]	1-1,000
ML [1]	…	…	…	…
ML [1]	.4 [3]	15 [5]	500 [4] (last ML)	1-1,000
ULS [2]	.2 [1]	4 [1]	200 [1] (first ULS)	1-1,000
ULS [2]	.2 [1]	4 [1]	300 [2]	1-1,000
ULS [2]	.2 [1]	4 [1]	400 [3]	1-1,000
ULS [2]	.2 [1]	4 [1]	500 [4]	1-1,000
ULS [2]	…	…	…	…
ULS [2]	.4 [3]	15 [5]	500 [4] (last ULS)	1-1,000

Note 1 – [category code in dataset].

Note 2 – Once dataset are ordered, a “*ID*” variable has been created (ML: 1 – 60,000 solutions; ULS: 1 – 60,000 solutions). See the *New dependent variables section*.

- **Raw data files**: the name of the file shows simulated sample size and magnitude of population factor loading. For example, “N200L020” is the file that contains raw data for $N = 200$ and $\lambda_{ik} = 0.20$. “CASE” column allows identifying the raw data of any estimated solution (each row of ML-ULS files).

3. Data generation

- **PRELIS 2** (Jöreskog & Sörbom, 1996b): 1,000 sample replications were simulated with the PRELIS program, using the same random seed. The variance-covariance matrix (**S**) and the correlation matrix (**R**) were computed for each sample.
  - **First step**: data file generation from population structures (*.DAT).
  - **Second step**: **S** (*.CM) and **R** (*.KM) matrices for each sample data generated in step 1.

4. Model estimation

- **LISREL 8.8** (Jöreskog & Sörbom, 1996a): CFA was conducted with the LISREL program for each **S** and **R** matrices. Convergence criterion: up to 250 iterations (IT=250).
    - **NOTE 1**: ULS is not a suitable method to estimate CFA models from **S** matrices since the size of the residuals depends on the scale of measurement of indicators, being more appropriate to use it from **R** (e.g., Jöreskog, Sörbom, Du Toit, & Du Toit, 2001). Then, ML was used with **S** matrices and ULS was used with **R** matrices.
    - **NOTE 2**: in a Monte Carlo simulation study, LISREL generates three types of files: *.PV contains estimated parameters, *.SV contains estimated standard errors, and *.GF contains several fit measures (Jöreskog & Sörbom, 1996b, p. 192).
- **Parameters estimates and standard errors** (*.PV and *.SV):
    - o **LX1.1 – LX15.1**: estimated value of parameter λ_{ik} (*factor loading*) for each *p*/*k* indicator in each LISREL solution. *STANDARDIZED*.
    - o **TD.1 – TD.15**: estimated value of parameter δ_{ik} (*measurement error*) for each *p*/*k* indicator in each LISREL solution. *STANDARDIZED*.
    - o **EX1 – EX15**: standard error of each *p*/*k* parameter λ_{ik}.
    - o **ETD1 – ETD15**: standard error of each *p*/*k* parameter δ_{ik}.
    - o **LXUN1.1 – LXUN15.1** (<u>ML estimation</u>): *UNSTANDARDIZED* λ_{ik} for each *p*/*k* indicator in each LISREL solution.
    - o **TDUN.1 – TDUN.15** (<u>ML estimation</u>): *UNSTANDARDIZED* δ_{ik} (*measurement error*) for each *p*/*k* indicator in each LISREL estimated model, replication or solution.
        - **NOTE 3**: for improve comparability, parameters λ_{ik} and δ_{ik} has been standardized after conducting CFA on **S** matrices (ML estimation). Variables LX(1.1 – 15.1) are standardized both in ML and ULS LISREL solutions.
        - **NOTE 4**: unstandardized estimated values of λ_{ik} and δ_{ik} ("LXUN" and "TDUN") are provide in separate file (UNSTANDARDIZED_parameters_ML SPSS/Excel file).

5. Model evaluation (*dependent variables*)

- **LISREL output**:
    - o **General information** (all types of files: *.PV, *.SV, and *.GF)**:**
        - *CASE*: sample replication number (1 to 1,000) for each experimental condition.
        - *CON* (Jöreskog & Sörbom, 1996b):
            - ⇒ **"0"** if iterations have converged and the *p*-value for χ^2 is in the interval .0005 ≤ p ≤ .9995.

- ⇒ **“1”** if iterations have not converged (non-convergent solution).
- ⇒ **“2”** if iterations have converged and the *p*-value for χ^2 is either $p < .0005$ or $p > .9995$. There are not CON = 2 solutions in this BBDD.

- *ADM* (Jöreskog & Sörbom, 1996b):
  - ⇒ **“0”** if the solution is admissible.
  - ⇒ **“1”** if the solution is not admissible.

    NOTE: a new variable (HEY, see the *New dependent variables* section) has been computed to identify parameter estimates that are not admissible (i.e., *Heywood* cases).

- **Parameter estimates and standard errors** (*.PV and *.SV): see *Model estimation* section.
- **Goodness-of-fit statistics** (*.GF)**:** see Jöreskog & Sörbom (1993, 1996a) and Marsh, Hau, & Grayson (2005).
  - ***gl***: Degrees of Freedom.
  - ***MFFCHI*** (<u>ML estimation</u>): Minimum Fit Function χ^2 with “*gl*” degrees of freedom.
  - ***MFFCHIp*** (<u>ML estimation</u>): Minimum Fit Function χ^2 with “*gl*” degrees of freedom (*p*-value).
  - ***NCHI*** (<u>ULS estimation</u>): Normal Theory Weighted Least Squares χ^2 with “*gl*” degrees of freedom.
  - ***NCHIp*** (<u>ULS estimation</u>): Normal Theory Weighted Least Squares χ^2 with “*gl*” degrees of freedom (*p*-value).
  - ***SBCHI***: Satorra-Bentler Scaled χ^2 with “*gl*” degrees of freedom. NOTE: SBCHI = 0 with continuous data (available for discrete data analysis).
  - ***SBCHIp***: Satorra-Bentler Scaled χ^2 with “*gl*” degrees of freedom (*p*-value). NOTE: SBCHIp = 1 with continuous data (available for discrete data analysis).
  - ***CorrCHI***: χ^2 with “*gl*” degrees of freedom corrected for Non-Normality. NOTE: CorrCHI = 0 with continuous data (available for discrete data analysis).
  - ***CorrCHIp***: χ^2 with “*gl*” degrees of freedom corrected for Non-Normality (*p*-value). NOTE: CorrCHIp = 1 with continuous data (available for discrete data analysis).
  - ***NCP***: Estimated Non-Centrality Parameter.
  - ***NCPi***: 90 % Confidence Interval for NCP (limit inferior).
  - ***NCPs***: 90 % Confidence Interval for NCP (limit superior).

- ***MFFV***: Minimum Fit Function Value.
- ***F0***: Population Discrepancy Function Value.
- ***F0i***: 90 Percent Confidence Interval for F0 (limit inferior).
- ***F0s***: 90 Percent Confidence Interval for F0 (limit superior).
- ***RMSEA***: Root Mean Square Error of Approximation.
- ***RMSEAi***: 90 Percent Confidence Interval for RMSEA (limit inferior).
- ***RMSEAs***: 90 Percent Confidence Interval for RMSEA (limit superior).
- ***RMSEA05***: *p*-value for Test of Close Fit (RMSEA > .05).
- ***ECVImod***: Expected Cross Validation Index for evaluated model.
- ***ECVImodi***: 90 Percent Confidence Interval for ECVI (limit inferior).
- ***ECVImods***: 90 Percent Confidence Interval for ECVI (limit superior).
- ***ECVIsat***: ECVI for saturated model.
- ***ECVIind***: ECVI for independence model.
- ***CHIind***: χ^2 for Independence Model (χ^2_{Null}) with "*gl_indep*" degrees of freedom. NOTE 1: "*gl_indep*" is not provide in *.GF file. It can be calculated as *p*/*k*(*p*/*k*-1)/2. NOTE 2: χ^2_{Null} *p*-value is not provide in *.GF file. It can be calculated by the statistical function between "*CHIind*" and "*gl_indep*" degrees of freedom (e.g., by a SPSS or Excel function). See the *New dependent variables* section.
- ***AICind***: Akaike Information Criterion for independence model.
- ***AICmod***: Akaike Information Criterion for evaluated model.
- ***AICsat***: Akaike Information Criterion for saturated model.
- ***CAICind***: Consistent Akaike Information Criterion for independence model.
- ***CAICmod***: Consistent Akaike Information Criterion for evaluated model.
- ***CAICsat***: Consistent Akaike Information Criterion for saturated model.
- ***RMR***: Root Mean Square Residual.
- ***SRMR***: Standardized RMR.
- ***GFI***: Goodness of Fit Index.
- ***AGFI***: Adjusted GFI.
- **PGFI**: Parsimony GFI.
- ***NFI***: Normed Fit Index.
- ***NNFI***: Non-Normed Fit Index (TLI).
- ***PNFI***: Parsimony NFI.
- ***CFI*** (ML estimation): Comparative Fit Index.
- ***IFI***: Incremental Fit Index.

- ***RFI*** (ML estimation): Relative Fit Index.
- ***CNHoelter*** (ML estimation): Critical *N* (Hoelter, 1983).

- **New *dependent variables***: variables that are calculated after conducting CFA (not provide in *.PV, *.SV, or *.GF files).
  - ***HEY***: Solutions with Heywood cases (this variable replaces ADM variable). LISREL 8 has a built-in-check on admissibility of the estimated solutions (see variable "*ADM*"). There is not enough information about this Heywood detection tool, and does not identify correctly this type of cases in all solutions. Variable "*HEY*" has been calculated as an alternative variable of admissibility: negative measurement errors ("*TD*") and factor loadings ("LX") < -1 or > 1 are code as 1 (otherwise – 0).
  - ***Tsolution***: Type of solution ("0" – Proper solutions, "1" – Improper solution). Improper solution can be a CON = 1 solution, a HEY = 1 solution, or both.
  - ***gl_indep***: Independence Model $p/k(p/k-1)/2$ degrees of freedom.
  - ***CHIindp***: χ^2 for Independence Model (χ^2_{Null}) with "*gl_indep*" degrees of freedom (*p*-value).
  - ***CONGR***: *Coefficient of Congruence* (C_k). This coefficient is computed as an index of factor similarity (see equation 1). C_k was initially proposed by Tucker (1951), and computes the discrepancy between population factor loadings (λ_{ik}) and estimated standardized factor loadings (λ^*_{ik}) for each indicator *i* of factor *k*, where *p* is the number of indicators per factor (*p*/*k*). C_k reflects a combined measure of good or poor parameter recovery of a given cluster of indicators. Lorenzo-Seva & Ten Berge (2006) have shown that congruence values in the range of .85 - .95 can be considered as "fair similarity" between λik and λ*ik, and values higher than .95 as "good similarity".

$$C_k = \frac{\sum_{i=1}^{p} \lambda^*_{ik} \lambda_{ik}}{\sqrt{(\sum_{i=1}^{p} \lambda^{*2}_{ik})(\sum_{i=1}^{p} \lambda^{2}_{ik})}} \qquad (1)$$

6. *Path diagrams* and matrix notation

(e.g., $p/k = 4$)

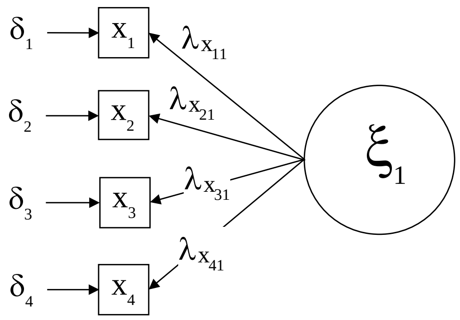

$$\begin{cases} \boldsymbol{\Sigma} = \boldsymbol{\Lambda\Phi\Lambda}' + \boldsymbol{\Theta} \\ \begin{pmatrix} x_1 \\ x_2 \\ x_3 \\ x_4 \end{pmatrix} = \begin{pmatrix} \lambda_{11} \\ \lambda_{21} \\ \lambda_{31} \\ \lambda_{41} \end{pmatrix} (\xi_1) + \begin{pmatrix} \delta_1 \\ \delta_2 \\ \delta_3 \\ \delta_4 \end{pmatrix}, \\ \boldsymbol{\Phi} = (\phi_{11}) = 1, \\ \boldsymbol{\Theta} = diag(\delta_1, \delta_2, \delta_3, \delta_4). \end{cases}$$

Simulated λ_{ik} = (.2, .3, .4)

Simulated δ_{ik} = (.96, .91, .84)

$x_i \sim N(0,1)$

7. *References*

Hoelter, J. W. (1983). The analysis of covariance structures: goodness-of-fit indices. Sociological Methods and Research, 11, 325–344. doi:10.1177/0049124183011003003

Jöreskog, K. G., & Sörbom, D. (1993). *LISREL 8: Structural equation modeling with the SIMPLIS command language*. Scientific Software International.

Jöreskog, K. G., & Sörbom, D. (1996a). *LISREL 8: User's reference guide*. Scientific Software International.

Jöreskog, K. G., & Sörbom, D. (1996b). *PRELIS 2: User's reference guide*. Scientific Software International.

Jöreskog, K. G., Sörbom, D., Du Toit, S. H. C., & Du Toit, M. (2001). *LISREL 8: new statistical features*. Scientific Software International.

Lorenzo-Seva, U., & Ten Berge, J. M. (2006). Tucker's congruence coefficient as a meaningful index of factor similarity. *Methodology*, *2*(2), 57-64.

Marsh, H. W., Hau, K., & Grayson, D. (2005). Goodness of fit in structural equation models. In A. Maydeu-Olivares & J. J. McArdle (Eds.), *Contemporary Psychometrics* (pp. 275–340). Psychology Press.

Tucker, L. R. (1951). A method for synthesis of factor analysis studies. *Personnel Research Section Report, 984*. Department of the Army, Washington, D.C.
